# Generation of myeloid-derived suppressor cells using prostaglandin E_2_

**DOI:** 10.1186/2047-1440-1-15

**Published:** 2012-09-28

**Authors:** Nataša Obermajer, Pawel Kalinski

**Affiliations:** 1 Departments of Surgery, University of Pittsburgh, Hillman Cancer Center, Pittsburgh, PA, 15213, USA; 2 Departments of Immunology, University of Pittsburgh, Hillman Cancer Center, Pittsburgh, PA, 15213, USA; 3 Infectious Diseases and Microbiology, University of Pittsburgh, Pittsburgh, PA, 15213, USA; 4 University of Pittsburgh Cancer Institute, University of Pittsburgh, Hillman Cancer Center, Pittsburgh, PA, 15213, USA; 5 Department of Surgery, University of Pittsburgh, Hillman Cancer Center, UPCI Research Pavilion, Room 1.46, 5117 Center Avenue, Pittsburgh, PA, 15213-1863, USA

**Keywords:** Cancer, COX2, Dendritic cells, Human, Immune dysfunction, Immunotherapy, Myeloid-derived suppressor cells, PGE_2_, Transplantation

## Abstract

Myeloid-derived suppressor cells (MDSCs) are natural immunosuppressive cells and endogenous inhibitors of the immune system. We describe a simple and clinically compatible method of generating large numbers of MDSCs using the cultures of peripheral blood-isolated monocytes supplemented with prostaglandin E_2_ (PGE_2_). We observed that PGE_2_ induces endogenous cyclooxygenase (COX)2 expression in cultured monocytes, blocking their differentiation into CD1a^+^ dendritic cells (DCs) and inducing the expression of indoleamine 2,3-dioxygenase 1, IL-4Rα, nitric oxide synthase 2 and IL-10 - typical MDSC-associated suppressive factors. The establishment of a positive feedback loop between PGE_2_ and COX2, the key regulator of PGE_2_ synthesis, is both necessary and sufficient to promote the development of CD1a^+^ DCs to CD14^+^CD33^+^CD34^+^ monocytic MDSCs in granulocyte macrophage colony stimulating factor/IL-4-supplemented monocyte cultures, their stability, production of multiple immunosuppressive mediators and cytotoxic T lymphocyte-suppressive function. In addition to PGE_2_, selective E-prostanoid receptor (EP)2- and EP4-agonists, but not EP3/1 agonists, also induce the MDSCs development, suggesting that other activators of the EP2/4- and EP2/4-driven signaling pathway (adenylate cyclase/cAMP/PKA/CREB) may be used to promote the development of suppressive cells. Our observations provide a simple method for generating large numbers of MDSCs for the immunotherapy of autoimmune diseases, chronic inflammatory disorders and transplant rejection.

## Biology of myeloid-derived suppressor cells

Dendritic cells (DCs) are key initiators and regulators of immune responses [1-3]. Therapeutic programming of DCs to suppress their function has been shown beneficial in autoimmunity and transplantation [4-6]. In contrast to DCs, suppressive macrophages [7] and myeloid-derived suppressor cells (MDSCs), originally shown to accumulate at the site of tumors, suppress the ability of CD8^+^ T cells to mediate effective responses against cancer cells, but can be beneficial in controlling autoimmune phenomena or transplant rejection [8-10].

MDSCs [10], important mediators of tumor-induced immune dysfunction and cancer progression [11], represent a heterogeneous population of immature myeloid cells (iMCs) involving precursors of macrophages, granulocytes, and DCs. MDSCs express CD34, common myeloid marker CD33, macrophage/DCs marker CD11b, and IL4Rα (CD124), but lack expression of the lineage (Lin) markers of DCs and other mature myeloid cells [10,12]. Human MDSCs are defined as CD33^+^Lin^-^HLA-DR^-/low^ cells. Recent studies demonstrate that monocytic MDSCs from patients with melanoma [13], prostate cancer [14], gastrointestinal malignancies [15], hepatocellular carcinoma [16,17] and glioblastoma [18] show a CD14^+^CD11b^+^HLA-DR^low^ phenotype while neutrophil-related immature (i)MDSCs present in peripheral blood show CD15 expression [10].

MDSCs express high levels of immunosuppressive factors, such as indoleamine 2,3-dioxygenase (IDO) [19,20], IL-10 [12], arginase [21,22], inducible nitric oxide synthase (iNOS, NOS2) [22], nitric oxide, and reactive oxygen species [23] and use these molecules to suppress T-cell responses [24,25]. Their induction of natural killer cell anergy and reduced cytotoxicity is arginase-independent [16] but depends on transforming growth factor β_1_[26]. PD-L1/B7-H1, induced on MDSCs [27,28], suppresses antigen-specific immunity via interaction with regulatory T cells (T_reg_) [27], enhanced T cell IL-10 expression and reduced IFN-γ production [28].

The presence of prostaglandin E_2_ (PGE_2_) at early stages of DC development was shown to suppresses the differentiation of human monocytes into functional T helper (Th)1-inducing CD1a^+^ DCs [29]. Additionally, PGE_2_ is needed for the development of tumor-associated suppressive macrophages [30-32]. Our two recent reports [33,34] demonstrate that PGE_2_ is both required and sufficient to redirect the differentiation of human dendritic cells into monocytic MDSCs. It also mediates the induction of MDSC-associated suppressive factors in human MDSCs [21] in a mechanism involving the establishment of a positive feedback loop between PGE_2_ and cyclooxygenase (COX)-2 [33], the key regulator of PGE_2_ production [35]. Additionally, PGE_2_ has been shown to enhance the numbers of MDSCs in mouse models and induce their expansion *ex vivo*[36-38].

## *In vitro* generation of myeloid-derived suppressor cells

Recent work in mice demonstrated that functional MDSCs can be generated *in vitro* from mouse embryonic stem cells and bone marrow hematopoietic stem cells, resulting in two subpopulations - CD115^+^Ly-6C^+^ (equivalent to the monocytic Gr-1^+^CD115^+^F4/80^+^ MDSCs found in tumor-bearing mice) and CD115^+^Ly-6C^-^ cells (resembling the granulocyte/macrophage progenitors) [37,39-41]. Adoptive transfer of these MDSCs prevented graft-versus-host disease mediated by alloreactive T cells. While granulocytic MDSCs may induce non-specific immune suppression and suppress the effector phase of the allogeneic immune response at an early stage, the monocytic MDSCs emerge as the key subset needed to promote T_reg_ development and to establish long-term antigen-specific tolerance [37,39-41]. Another source of MDSCs is the bone marrow, which harbors a large reservoir of MDSCs. Recent studies have demonstrated an efficient growth factor/cytokine (granulocyte macrophage colony stimulating factor (GM-CSF) + G-CSF or GM-CSF + IL-6 or IL-13)-induced expansion of MDSCs populations *in vitro*, utilizing bone marrow cells from either mice or human sources [42,43] to generate IL4Rα^+^ MDSCs. In mice these cells were able to impair the priming of CD8^+^ T cells, and enabled long-term acceptance of pancreatic islet allografts [43]. Furthermore, bone marrow progenitor cells can be induced by lipopolysaccharide to develop into CD11b^+^Gr1^int^F4/80^+^ cells that, when adoptively transferred, suppressed allergen-induced airway inflammation in recipient mice [44]. Due to the massive accumulation of MDSCs in the spleens of tumor-bearing mice, the spleen is considered to be a reservoir of MDSCs and their precursors [45]. The drawback of these reported initiatives to develop MDSC-based therapeutic strategies is the lack of a reliable source of MDSCs.

For human treatment regimens the control of MDSCs *in vitro* by manipulating recipient myelomonocytic precursor cells appears most applicable. While there are low frequency and total numbers of MDSCs in peripheral blood (approximately 5% of cells in healthy subjects), peripheral blood constitutes a very convenient source of myelomonocytic precursor cells for MDSC generation. Apart from the recently described cytokine regimens that showed the feasibility of *in vitro* expansion of blood-isolated MDSCs populations [46] the induction of human MDSCs has been proven a feasible *in vitro* approach for the generation of CD14^+^HLADR^neg/low^ MDSCs by differentiation of isolated CD14^+^ cells in the presence of IL-4 + GM-CSF and tumor-derived microvesicles [46]. Alternatively, functional MDSCs can be induced in peripheral blood mononuclear cell (PBMC) cultures supplemented with several cytokine induction combinations, produced by tumor cell lines [47].

Our current data provides evidence for the feasibility of generating large numbers of monocytic MDSCs for the immunotherapy of autoimmune and inflammatory diseases, or transplant rejection by using a single common determining factor - PGE_2_, a common inflammation-associated master regulator of immune responses - that can redirect the development of CD1a^+^ DCs to CD14^+^CD33^+^CD34^+^ monocytic MDSCs [48].

## Efficient generation of human myeloid-derived suppressor cells using prostaglandin E_2_

The development of functional MDSCs requires the inhibition of development of immunostimulatory antigen presenting cells and concomitant induction of suppressive functions [8]. The expansion of iMCs can be induced by factors such as GM-CSF, IL-6, or vascular endothelial growth factor [24,49-51]. The upregulation of MDSC-associated immunosuppressive factors and establishment of their immunosuppressive function can be induced by such factors as IL-1β, IFNγ, PGE_2_, or Toll-like receptor ligands [8]. While the above MDSC-activating factors have apparently diverse character and functions, they all share the ability to induce COX2 expression and PGE_2_ production [52-54], suggesting the key role of COX2 and PGE_2_ in MDSCs development.

**Figure 1:**
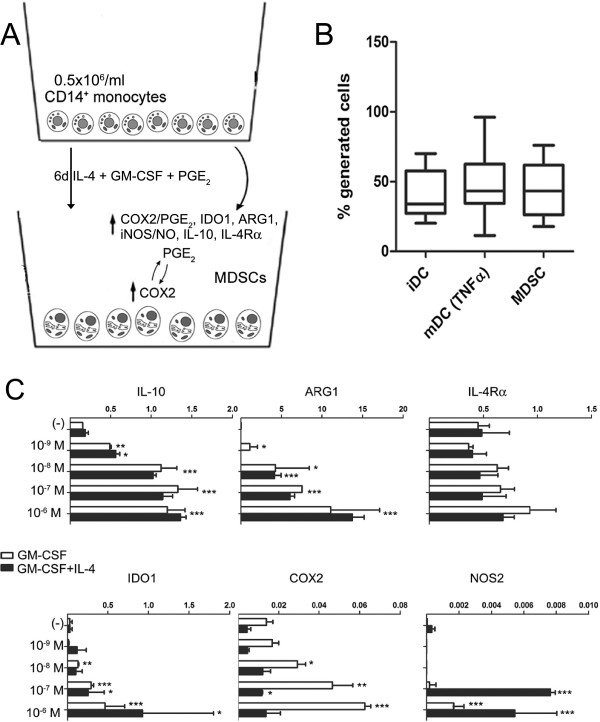
**Prostaglandin E**_**2**_**-induced positive cyclooxygenase 2-prostaglandin E**_**2**_**-E-prostanoid receptor 2/4 feedback loop allows for *ex vivo *generation of high numbers of myeloid-derived suppressor cells and their functional stability.** (**A**) Prostaglandin E_2_ (PGE_2_) (via E-prostanoid receptor (EP)2- and EP4-dependent signals) drives the early induction of cyclooxygenase (COX)2 in local myeloid cells (monocytes, macrophages, immature dendritic cells (iDCs)), promoting their production of suppressive factors (indoleamine 2,3-dioxygenase (IDO)1, IL-10, arginase 1, nitric oxide synthase (NOS)2, and PGE_2_ itself (current data and [48]), and acquisition of suppressive functions [48]*.* These processes are further amplified by the *de novo* production of endogenous PGE_2_, now produced at high levels by myeloid-derived suppressor cells (MDSCs) themselves, thereby creating a positive feedback loop leading to persistence of MDSCs. The key role of the EP2- and EP4-mediated COX2-PGE_2_ feedback to control multiple aspects of MDSCs function provides convenient targets to generate MDSC-associated immune regulation in tolerogenic therapies. (**B**) PGE_2_ induces high numbers of MDSCs (48.6%), with yields similar to iDCs (40.2%) and TNF-α matured DCs (36.9%). Percentages indicate the yields of the cells generated in day 6 monocyte cultures performed in the presence of granulocyte macrophage colony stimulating factor (GM-CSF) and IL-4 in the absence of PGE_2_ (iDC, CD1a^+^ DCSIGN^+^CD14^-^CD80^-^CD83^-^) or presence of PGE_2_ (MDSCs, CD1a^-^DCSIGN^-^CD14^+^CD33^+^CD34^+^CD80^-^CD83^-^) and after additional 48 h maturation of iDC with TNF-αmDC, (CD1a^+^ DCSIGN^+^CD14^-^CD80^+^CD83^+^). Bars present data (mean ± s.d.) from 12 different experiments with different donors. (**C**) Dose-dependent induction of immunosuppressive factors IL10, IDO1, IL4Rα and COX2 in PGE_2_-induced MDSCs, generated in the presence or absence of IL-4 (relative mRNA levels normalized for hypoxanthine phosphoribosyltransferase 1 and expressed as fold increase (2^-ΔCT^), where ΔCT = CT _(Target gene)_ - CT _(HPRT1)_). Bars present data (mean ± s.d) of a single representative experiment with different donors. *P <0.05, **P <0.01, ***P <0.001, statistically significant differences relative to medium alone.

Peripheral blood-derived monocytes provide a convenient source of cells for cellular therapy due to their relative abundance in the circulation. We used peripheral blood PBMCs, obtained from the blood of healthy donors (Central Blood Bank of Pittsburgh, PA) using lymphocyte separation medium, to isolate monocytes by positive magnetic selection using the CD14^+^ isolation kit (EasySep Isolation kit; Stem Cell Tech, Vancouver, Canada). Monocytes were cultured for 6 days in 12 or 24-well plates at 5 × 10^5^ cells per well in rhuGM-CSF and IL-4 (both 1000 U/ml; gifts from Schering Plough, Kenilworth, NJ), with 10^-6^ M PGE_2_ (PGE_2_-induced MDSCs, Sigma, St Louis, MO, USA) (Figure 1A). Alternatively, the E-prostanoid receptor (EP)2 agonist Butaprost (10 μM, Sigma) and the EP4 agonist CAY10598 (10 nM, Cayman Chemical, Ann Arbor, MI, USA) were used to generate MDSCs. EP2 and EP4 are the two subtypes of the G protein-coupled receptor, signaling of which is coupled to a rise in cAMP concentration [55]. As shown in Figure 1B, the yield of PGE_2_-induced MDSCs (CD1a^-^DCSIGN^-^CD14^+^CD33^+^CD34^+^CD80^-^CD83^-^) was similar to the yield of iDCs (CD1a^+^ DCSIGN^+^CD14^-^CD80^-^CD83^-^) and TNF-α-matured (rhuTNFα, 50 ng/ml, Strathmann Biotech, Germany) DCs (CD1a^+^ DCSIGN^+^CD14^-^CD80^+^CD83^+^).

The differentiation of monocytes into functional CD1a^+^ DCs could be redirected into CD1a^-^CD14^+^CD80^-^CD83^-^ MDSCs by their exposure to PGE_2_ only at early stages of DC development (that is, from day 0, PGE_2_^d0^) [29] but not at later time points (that is, at day 6, PGE_2_-conditioned DCs^d6^).

While the immunosuppressive phenotype of the PGE_2_-induced MDSCs proved to be PGE_2_ concentration-dependent (Figure 1C) [29], it was independent of the presence of IL-4, indicating a key role for PGE_2_, but not for IL-4, in inducing MDSCs.

Exposure to PGE_2_ induced the expression of endogenous COX2 in differentiating monocytes, leading to the establishment of a PGE_2_-COX2-mediated positive feedback loop, and the induction of IDO1, NOS2, IL-10, or IL-4Rα - the typical MDSC-associated factors (Figure 1C). PGE_2_-induced cells displayed a suppressive phenotype, marked by the expression of inhibitory molecules - inhibitory receptor Ig-like transcript (ILT)2, ILT3, ILT4 and programmed cell death 1 ligand 1 (previously implicated in the suppressive functions of myeloid cells [27,28]), produced the immunosuppressive factors IDO1, IL10 and PGE_2_ and exerted suppressive functions, blocking the proliferation and development of CD8^+^ T cells into granzyme B (GrB)^high^ cytotoxic T lymphocytes [33].

Additionally, PGE_2_ induced a uniform expression of high levels of CXCR4 [34], typically present on MDSCs from cancer-bearing individuals [56], and strong migratory responsiveness to CXCL12 [34].

## Therapeutic potential of *ex vivo* induced myeloid-derived suppressor cells

Anti-inflammatory activity of MDSCs in a variety of physiological settings and their therapeutic promise in transplantation [57] suggest that these cells may provide a novel cell-based immunotherapy in transplantation [40,58] and autoimmune diseases [59].

While the spontaneously arising endogenous MDSCs present in many forms of autoimmune diseases appear to be defective and ineffective in controlling the disease (reviewed in [60]), it was shown that adoptive transfer of MDSCs can limit autoimmune pathology [61-63], providing a rationale for the development of methods to expand or induce MDSCs *ex vivo*.

Transfer of MDSCs can prevent graft-versus-host disease [42], and prolong the survival of allo-skin [64] and allo-kidney transplants [65], and play an essential role in an allogeneic cardiac transplantation model [57]. Adoptively transferred MDSCs, isolated from synegeic tumor-bearing mice, can prevent the onset of type 1 diabetes in non-obese diabetic mice [63] and ameliorate the symptoms of inflammatory bowel disease [59]. In a mouse model of alopecia, adoptively transferred MDSCs have been shown to promote partial restoration of hair growth [62].

From the therapeutic standpoint, it is important to identify central regulatory pathways that maintain the suppressive functions of MDSCs mediated by different suppressive molecules (arginase 1 [42], ILT-2 [66], heme-oxygenase (HO-1) [64], and iNOS [65]). Our data [48,67] - showing that the exposure of differentiating monocytes to PGE_2_ (and the establishment of a positive feedback between PGE_2_ and COX2) is both required and sufficient for MDSC stability and their ability to produce all MDSC-associated suppressive mediators and suppress CD8^+^ T cell function [48] - provides evidence for a feasible and clinically compatible method of generating suppressive cells for immunotherapeutic purposes.

## Conclusions

Due to their ability to suppress T cell responses in multiple diseases [65,68,69], MDSCs represent a promising population of cells for use in tolerogenic therapies. Our recent observations demonstrating the feasibility of using PGE_2_ to promote the development of MDSCs from monocytic precursors provide a clinically feasible system of generating large numbers of MDSCs *ex vivo*, facilitating the development of new therapies for autoimmune diseases and transplant rejection.

## Abbreviations

COX: Cyclooxygenase; DC: Dendritic cell; EP: E-prostanoid receptor; GM-CSF: Granulocyte macrophage colony stimulating factor; GrB: Granzyme B; HO-1: Heme-oxygenase; IDO: Indoleamine 2,3-dioxygenase; IFN: Interferon; IL: Interleukin; ILT: Inhibitory receptor Ig-like transcript; iMC: Immature myeloid cells; iNOS: Inducible nitric oxide synthase; Lin: Lineage; MDSC: Myeloid-derived suppressor cell; NOS: Nitric oxide synthase; PBMC: Peripheral blood mononuclear cell; PGE_2_: Prostaglandin E_2_; Th: T helper; T_reg_: Regulatory T cells.

## Competing interests

The authors declare that they have no competing interests.

## Authors’ contributions

NO and PK conceived the work and wrote the manuscript. Both authors read and approved the final manuscript.
